# Polarization-Insensitive, High-Efficiency Metasurface with Wide Reception Angle for Energy Harvesting Applications

**DOI:** 10.3390/s25020429

**Published:** 2025-01-13

**Authors:** Abdulrahman Ahmed Ghaleb Amer, Nurmiza Othman, Mohammed M. Bait-Suwailamn, Syarfa Zahirah Sapuan, Ali Ahmed Ali Salem, Adeb Salh

**Affiliations:** 1 Faculty of Electrical and Electronic Engineering, Universiti Tun Hussein Onn Malaysia (UTHM), Batu Pahat 86400, Johor, Malaysia; aag2014ye@gmail.com (A.A.G.A.); nurmiza@uthm.edu.my (N.O.); syarfa@uthm.edu.my (S.Z.S.); 2 Communication and Information Research Center, Sultan Qaboos University, Muscat 123, Oman; 3 Department of Electrical and Computer Engineering, Sultan Qaboos University, Muscat 123, Oman; 4 School of Electrical Engineering, Universiti Teknologi MARA (UITM), Shah Alam 40450, Selangor, Malaysia; alisalem@uitm.edu.my; 5 Faculty of Information and Communication Technology, University Tunku Abdul Rahman (UTAR), Kampar 31900, Perak, Malaysia; adebali@utar.edu.my

**Keywords:** electromagnetic energy harvesting, metasurface, polarization-insensitive, wide-angle, higher conversion efficiency

## Abstract

This research presents an innovative polarization-insensitive metasurface (MS) harvester designed for energy harvesting applications at 5 GHz, capable of operating efficiently over wide reception angles. The proposed MS features a novel wheel-shaped resonator array whose symmetrical structure ensures insensitivity to the polarization of incident electromagnetic (EM) waves, enabling efficient energy absorption and minimizing reflections. Unlike conventional designs, the metasurface achieves near-unity harvesting efficiency, exceeds 94% under normal incidence, and maintains superior performance across various incident angles for TE and TM polarizations. To validate the design, a 5 × 5-unit cell array of the MS structure was fabricated and experimentally tested, demonstrating excellent agreement between simulation and measurement results. This work significantly advances metasurface-based energy harvesting by combining polarization insensitivity, wide-angle efficiency, and high absorption, making it a compelling solution for powering wireless sensor networks in next-generation applications.

## 1. Introduction

The emergence of 5G technology has revolutionized connectivity, offering faster speeds, reduced latency, and increased capacity. As a result, it has facilitated the widespread adoption of low-power intelligent devices, such as IoT devices, sensors, and implantable medical devices. These compact and energy-efficient devices are now integral to various aspects of our lives. Furthermore, the technology enables wireless power supply to these devices through energy harvesting, a process that captures electromagnetic (EM) energy from the environment and converts it into DC power [1,2,3,4]. The EM energy harvesting in the microwave regime has garnered significant attention due to its advantageous characteristics, such as low cost, compact size, and potential for long-range power transfer [5]. Metamaterials represent cutting-edge technologies in manipulating EM waves, providing exceptional control over wave propagation, reflection, and absorption. These specially designed materials, distinguished by their subwavelength structural features, exhibit distinct EM properties not observed in natural materials, opening up new possibilities for innovative applications in energy harvesting [6,7].

Metasurface (MS) is a 2-D metamaterial structure consisting of subwavelength elements arranged periodically within a specific area with a thickness less than the wavelength of the EM waves it interacts with [8]. MSs offer unique capabilities in manipulating EM fields, making them suitable for various applications such as antenna design [9,10], cloak [11], absorber [12,13,14,15,16,17,18,19], imaging [20], wireless power transfer (WPT), and energy harvesting [21,22,23,24,25]. Recently, MSs have gained attention as energy collectors for energy harvesting applications, replacing conventional antennas due to their advantageous properties, including multi-polarization and wide coverage angle. The performance of an MS critically depends on the geometry of the structure and the periodic arrangement of unit cells. The metal–dielectric–metal (MDM) MS structure is exceptionally efficient for energy harvesting and can be adjusted to enhance harvesting effectiveness at particular frequencies. The top metallic resonator is connected to the ground plane through an optimal resistor load through a harvesting port (Via), ensuring efficient energy transfer and impedance matching while minimizing energy loss and maximizing efficiency [26,27]. The MS-based energy harvesting performance has been optimized with various works, including improving the harvesting bandwidth [28,29], enhancing the harvesting efficiency [27], and wide-band [28,30]. A rectifying MS array where each array element was connected to a rectifier circuit was proposed in [31,32,33]. Furthermore, to increase the overall conversion harvesting efficiency, the MS array elements were connected to a single rectifier circuit using a corporate feed network [34,35,36,37]. In addition, multi-band MS harvesters were designed to capture microwave radiation from multiple sources across different frequency ranges [38,39,40].

To optimize energy harvesting, MS-based energy harvesting is equipped with multi-polarization capabilities, enabling it to capture EM waves irrespective of their polarization [41,42,43,44]. Polarization-insensitive MS harvesters based on the split ring resonator (SRR) were reported in [44,45,46,47], where each unit cell of the MS array is connected to optimal resistor loads using four vias. The presence of four vias adds complexity and manufacturing challenges and distributes the received EM power into four loads, complicating subsequent power synthesis. In [48], polarization-insensitive MS-based energy harvesting was attempted using a butterfly-shaped closed-ring resonator. However, a higher harvesting efficiency was observed with a resistor load of approximately 3 KΩ, which complicated the design of the power-combining network and restricted its applicability in real-world harvesting systems. Furthermore, in some recent work, MS structures loaded with resistor loads placed between two sections of the resonator were designed for energy-harvesting applications [49,50,51]. It is worth noting that, in these configurations, the resistors were not connected to the ground. As a result, replacing them with rectifying circuits or combining networks presents a challenge.

The main contribution of this work can be concluded as follows:

- Design and analysis of a compact, wide-angle, polarization-insensitive MS collector for energy harvesting application at the 5 GHz band.
- A near-unity harvesting efficiency exceeding 94% is achieved regardless of the polarization angle of the incident wave under normal incidence.
- Achieving a higher harvesting efficiency at different incident angles up 60° and 75° for TE and TM polarization, respectively.

This study presents a polarization-insensitive and wide-angle metasurface structure comprising an array of symmetric wheel resonators designed to resonate at 5 GHz for energy harvesting applications. The proposed MS harvester has two resistor loads representing the input impedance of the power-combining circuit in the complete harvesting system. Simulation results show that the proposed MS can efficiently absorb incoming EM wave power and deliver the maximum absorbed energy to the loads with a near-unity harvesting efficiency (exceeding 90%), regardless of the polarization of the incident wave under normal incidence. Furthermore, it can capture incident EM power and deliver it to the loads with higher harvesting efficiency under various incident angles. Each unit cell of the proposed MS array has only two terminals, simplifying the power-combining network, in contrast to designs in [44,45,46], where each cell is connected to four terminals. Additionally, the maximum harvesting efficiency is achieved at an impedance load of 50 Ω, whereas in [48], the resistance value of the terminals is 3 KΩ, requiring a more complex power-combining network. It is important to emphasize that, in this research, similar to early works on the design of harvesters [38,39,41,43,45,47], the loss resulting from the power combining network and rectifying circuits has not been considered.

## 2. Metasurface Unit Cell Design

**Figure 1:**
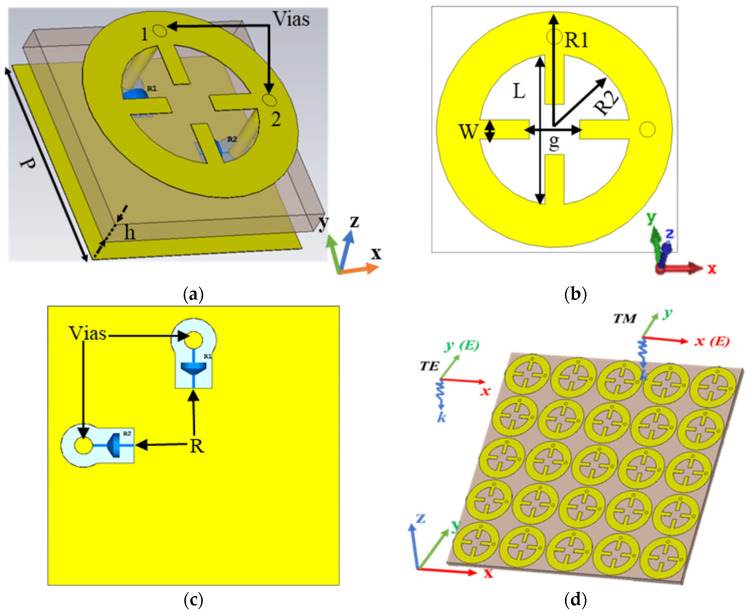
Geometry of the MS unit cell structure: (**a**) 3D view, (**b**) top view, (**c**) bottom view, and (**d**) schematic diagram of the MS array.

In this study, the MS unit cell is designed based on a wheel-shaped resonating structure, as depicted in Figure 1. The design incorporates an MDM structure, with the top metallic layer comprising a circular ring resonator to effectively interact with incident electromagnetic fields, while the cross-shaped slots enhance resonance and impedance matching. The bottom metallic layer serves as a ground plane. A dielectric substrate made of Rogers RO4003C with a dielectric constant of 3.55, tangent loss of 0.0027, and thickness of h = 1.524 mm is utilized to minimize energy loss. Furthermore, copper with a conductivity of 5 × 10^7^ S/m and thickness of 35 µm is used to fabricate the top metallic resonator, vias, and ground plane. Two vias are positioned on the two sides of the wheel-shaped resonator. Two annular gaps are opened around the vias at the ground plane to accommodate a load resistance of 50 Ω. 

Two resistor loads connect the metallic resonator to the ground plane through these vias. This arrangement ensures that the induced current can smoothly deliver to the load, allowing the top metallic resonator to receive incident waves at different incident and polarization angles. The wheel-shaped resonator structure is chosen for its simplicity and compact size, making it ideal for integrating energy harvesters into small electronic devices or wearable technology. Additionally, its rotational symmetric design makes it polarization-insensitive. Moreover, it offers enhanced EM coupling, leading to high efficiency at the desired frequency. The symmetrical wheel-shaped resonator facilitates large-angle performance, ensuring consistent interaction with electromagnetic waves across various angles. The inclusion of cross-shaped slots and a low-loss dielectric substrate further enhances field confinement and stability, preserving consistent resonance over a broad range of incident angles. The optimized parameters of the proposed MS unit cell harvester are listed in Table 1.

**Figure 2:**
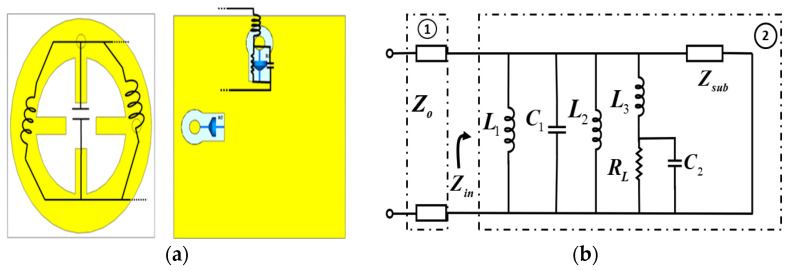
Equivalent RLC circuit model for MS unit cell. (**a**) A circuit schematic and (**b**) an equivalent circuit from the unit cell. The parameters are $L_{1}=1.24\;\mathrm{nH}$, $L_{2}=2.14\;\mathrm{nH}$, $L_{3}=4.28\;\mathrm{nH}$, $C_{1}=1.55\;\mathrm{pF}$, $C_{2}=0.4\;\mathrm{pF}$ and $R_{L}=50\;\Omega$.

The equivalent RLC circuit model is presented to analyze the absorption mechanism of the proposed MS structure, as shown in Figure 2. The equivalent circuit includes two sections. Section 1 represents the free space incident wave, which had an impedance of $Z_{o}=377\;\Omega$. Section 2 is a metasurface structure. The input impedance $\left(Z_{in}\right)$ of the proposed MS harvester can be described as

(1) $$Z_{in}=(R_{L}+j\omega L_{1}+\frac{1}{j\omega C_{1}}+j\omega L_{2}+j\omega L_{3}+\frac{1}{j\omega C_{2}})||Z_{sub}$$

where $L_{1}$, $L_{2}$, $C_{1}$ represent the equivalent inductance and capacitance of the top metallic wheel resonator and the split (g). $L_{3}$ represents the inductance of the metallic ground plane and vias. R is the harvesting load and $C_{2}$ is a capacitance between the via and ground plane. The substrate between the MS resonator and the ground plane can be modeled as a transmission line with an impedance of $Z_{sub}=Z_{o}/\sqrt{\varepsilon_{r}}$. The lumped element values in the equivalent circuit can be estimated using the microstrip line model [52]. Then, the estimated values for effective L, C, and R are optimized using the tuning function in the Keysight Advanced Design System (ADS) simulator.

## 3. Results and Discussion

### 3.1. Impact of Structure Parameters

- Normal incident analysis

The functionality of the MS unit cell was validated through numerical simulations conducted with CST Microwave Studio. In these simulations, periodic boundary conditions were applied to represent the unit cell structure as part of a larger construct using transverse electromagnetic (TEM) excitation. The unit cell boundary conditions were applied along the *x*- and *y*-axes, while open boundary conditions were implemented along the *z*-axis. Floquet ports were utilized in both TE and TM modes to facilitate excitation, with a plane wave incident along the *z*-axis.

**Figure 3:**
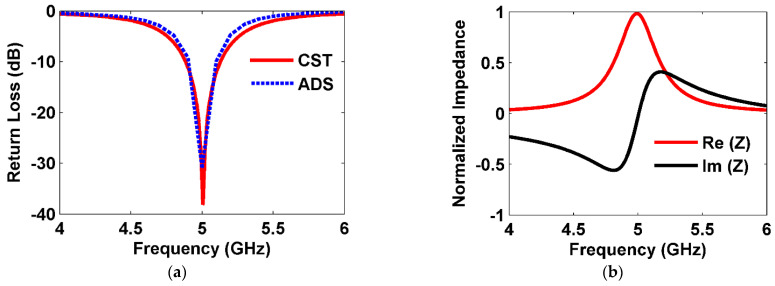

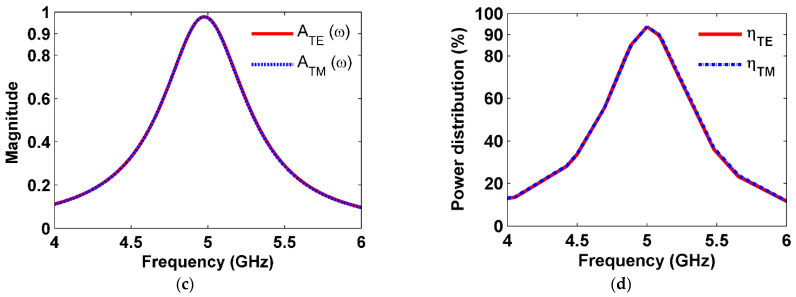
(**a**) S-parameter, (**b**) input impedance, (**c**) absorption at TE and TM polarization, and (**d**) efficiency at TE and TM polarization under normal incidence.

The return loss $\left(S_{11}\right)$ of the proposed MS unit cell was calculated using CST Microwave Studio and compared with the results obtained from aligned EM simulation software Advanced Design System (ADS) 2020, as shown in Figure 3a. From Figure 3a, the $S_{11}$ parameter was lower than −30 at normal incidence, indicating optimal performance with the minimal reflection of incident electromagnetic waves in free space. The figure compares the return loss (S11) results obtained from CST and ADS simulations, demonstrating a strong correlation at the resonance frequency of 5 GHz. The CST simulation, which employed 3D full-wave modeling, yielded sharper and more detailed results, whereas the ADS simulation, based on circuit-level modeling, simplified EM effects and resulted in a slightly broader dip. While there were minor discrepancies outside the resonance range, the findings effectively validate the design, emphasizing a trade-off between the accuracy of CST and the efficiency of ADS. The symmetrical design of the proposed MS unit cell made it insensitive to polarization. Therefore, the parameter for TE polarization was calculated. In addition, the normalized input impedance of the MS unit cell, terminated with 50 Ω resistor loads, was analyzed, as depicted in Figure 3b. At the resonant frequency of 5 GHz, the real part of the normalized impedance almost equaled 1, indicating a strong impedance match between the MS unit cell and free space. This resulted in efficient absorption of the incident wave, especially when 50 Ω resistor loads terminated the unit cell.

To verify the polarization insensitivity for the proposed MS unit cell, the absorption ratio under normal incidence for TE and TM polarization is calculated as follows:

(2) $$A(\omega )=1-|S_{11}(\omega ){|}^{2}-|S_{21}(\omega ){|}^{2}$$

where $S_{11}(\omega )$ and $S_{21}(\omega )$ are the reflection and transmission coefficients.

The back metallic layer of the structure acts as a physical barrier, effectively preventing transmission and resulting in an almost zero transmission coefficient. So, the absorptivity can be described as:

(3) $$A(\omega )=1-|S_{11}(\omega ){|}^{2}$$

The minimum reflection can be achieved when the impedance of the MS structure is well-matched with the impedance of free space, resulting in maximizing the absorptivity.

Figure 3c illustrates the absorption ratios for the MS unit cell under TE and TM polarizations. It achieves over 98% absorption peaks for both TE and TM polarizations at 5 GHz under normal incidence.

However, it is not enough to just reduce reflected waves; transferring the maximum absorbed power to the resistor load is also important for effectively collecting and reusing the incident EM waves. Therefore, the efficiency of power transmission to the resistor load can be calculated as follows:

(4) $$\eta =\frac{P_{Load}}{P_{Incident}}$$

where $P_{Load}$ and $P_{Incident}$ represent the power delivered to the load and power incident into the MS area. Figure 3d illustrates the conversion efficiency of the proposed MS unit cell for TE and TM polarizations. A higher harvesting efficiency peak of about 94% is achieved for both TE and TM polarizations under normal incidence.

**Figure 4:**
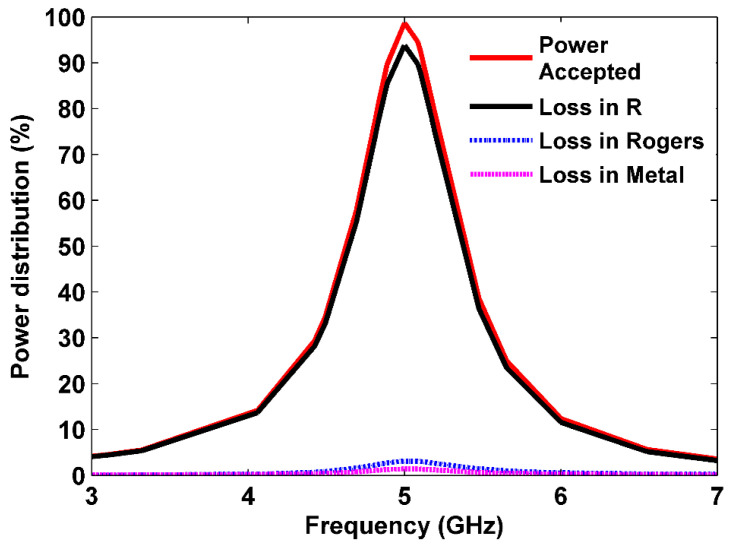
Power distribution in the cell.

The power loss was thoroughly studied to assess the energy harvesting capabilities of the proposed design. A full-wave simulation was performed to investigate the distribution of dissipated power within the unit cell across a wide frequency range. Figure 4 illustrates the power absorbed by the MS and the power losses in the resistor load, metal, and dielectric substrate (Rogers) when the incident EM waves propagated along the *z*-axis. This provided comprehensive insight into the distribution and dissipation of power within the unit cell. The power losses in the proposed MS unit cell were analyzed using CST Microwave Studio with a frequency-domain solver. Floquet ports with a fixed incident power value of $P_{incident}=0.5\;\mathrm{Watt}$ were used to excite the MS design. The simulation results for TE mode are provided because the MS unit cell was symmetrical and insensitive to polarization. As shown in Figure 4, the MS structure absorbed the most incident power, which was mainly concentrated in the resistor loads, achieving approximately 94% at 5 GHz. The power dissipated into the metal, and Rogers substrate material was almost negligible. This indicates that the proposed MS structure effectively collected the incident power.

**Figure 5:**
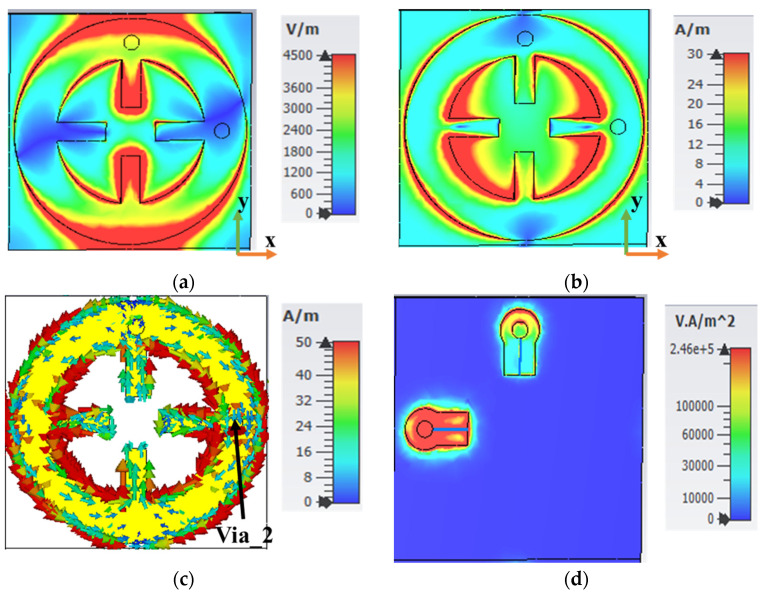
(**a**) E-field, (**b**) H-field, (**c**) current surface, and (**d**) power flow at 5 GHz for TE polarization under normal incidence.

**Figure 6:**
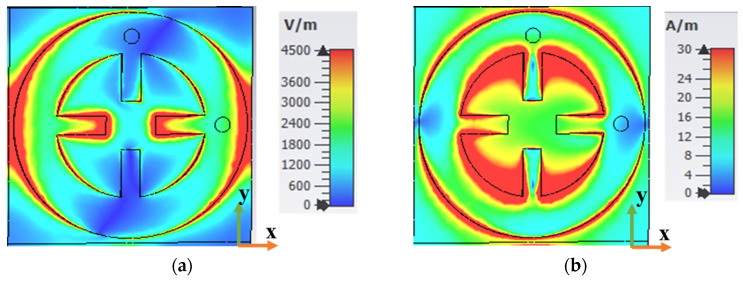

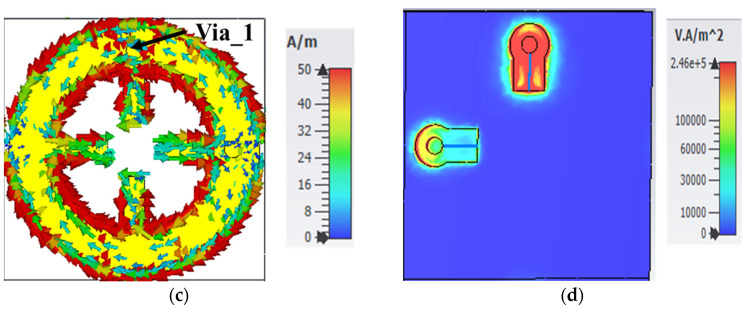
(**a**) E-field, (**b**) H-field, (**c**) current surface, and (**d**) power flow at 5 GHz for TM polarization under normal incidence.

To demonstrate the polarization-insensitive abilities of the MS structure, analysis of its electric field (E-field), magnetic field (H-field), surface current, and flow power for both TE and TM polarization at the resonance frequency of 5 GHz was conducted. Full-wave electromagnetic simulations were conducted in CST Microwave Studio for both TE and TM polarizations. The MS unit cell was analyzed under periodic boundary conditions along the *x*- and *y*-axes to simulate an infinite array. A plane wave with normal incidence excited the MS structure along the *z*-axis. The results of this analysis are presented in Figure 5 and Figure 6.

Figure 5 illustrates the electrical properties of the MS unit cell at 5 GHz for TE polarization under normal incidence. The electric field distribution in Figure 5a reveals that the EM resonance primarily occurred along the *y*-axis of the unit cell, with a clear accumulation of charge along this axis that then passed through the harvesting port (via) to the grounded resistor load (R1). The magnetic field (H-field) distribution, as depicted in Figure 5b, was more concentrated along the *x*-axis. The current surface distribution at 5 GHz for normal incidence is shown in Figure 5c, which reveals a current flow on both the inner and outer sides of the unit cell. This is evident from the intense red coloration observed across the wheel resonator. However, the magnitude of the electric current was more concentrated on the inner side of the wheel resonator than on the outer side. Additionally, the surface current was antiparallel at the top resonator and flowed through the via hole (Via_2) to dissipate into the resistor load (R2). The power distribution at the resonant frequency of 5 GHz is shown in Figure 5d. The power absorbed by the MS unit cell was primarily concentrated in the resistor load (R2), making the proposed MS structure highly suitable for energy harvesting applications.

Figure 6 displays the electrical properties of the proposed MS structure at 5 GHz for TM polarization with normal incidence, including E-field, H-field, current surface, and power flow. As shown in Figure 6a, the E-field distribution was most concentrated along the *x*-axis, then flowed through the via hole to the resistor load (R2). The H-field was distributed along the resonator, and it was more concentrated on the inner side of the wheel resonator, as seen in Figure 6b. Accordingly, Figure 6c shows the current surface distribution. It is clear that the current surface was antiparallel at the top resonator and then flowed through the harvesting hole (Via_1) into the resistor load (R1). Finally, the power flow distribution at 5 GHz is shown in Figure 6d. It is noted that the most power absorbed by the top resonator was delivered to the resistor load (R1), resulting in high harvesting efficiency.

**Figure 7:**
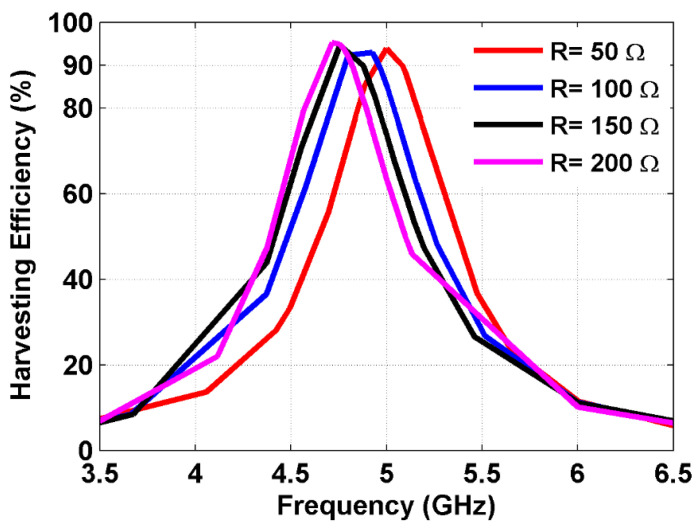
Harvesting efficiency at different resistor load values.

For additional verification, the conversion efficiency of the proposed MS structure in capturing EM power was assessed by adjusting the resistor load values. Figure 7 presents the harvesting efficiency of the proposed MS harvester as the resistor load value was varied from 50 Ω to 200 Ω. It is evident that an increase in the resistor load value shifted the harvesting efficiency peak to the lower frequency band due to the increase in the inductive and capacitive reactance. The maximum harvesting efficiency at 5 GHz was obtained when the resistor load value equaled 50 Ω, indicating optimal impedance matching between the MS harvester and free space.

B. Polarization angle analysis

**Figure 8:**
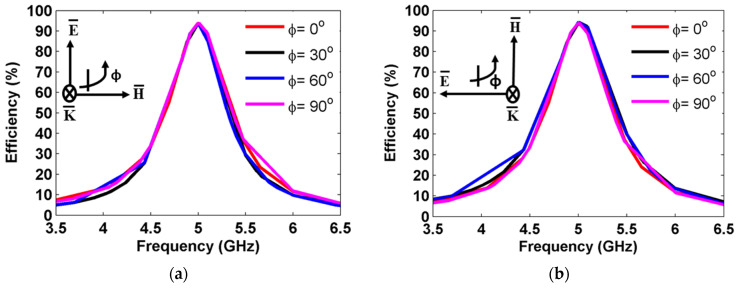
Harvesting efficiency for different polarization angles for (**a**) TE polarization and (**b**) TM polarization.

The polarization-insensitive behavior of the proposed MS structure was analyzed by varying the polarization angle (ϕ). Figure 8 shows the harvesting efficiency for the proposed MS array at normal incidence ($\theta_{inc}$ = 0°) when the polarization angle varied from 0° to 90° for both TE and TM polarizations. The harvesting efficiency was examined and calculated using Equation (4). As shown in Figure 8, the harvesting efficiency peak of 94% was achieved at various polarization angles up to 90° for both TE and TM polarizations under normal incidence. Furthermore, the proposed MS harvester maintained a consistent harvesting efficiency even with changes in polarization angles, showing that the MS structure is polarization-independent.

C. Incident angle analysis

**Figure 9:**
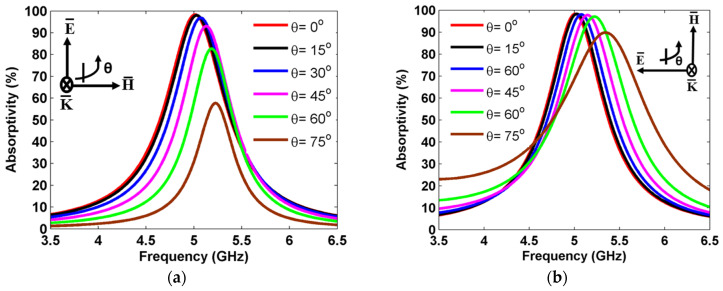
Absorptivity for different incident angles: (**a**) TE polarization and (**b**) TM polarization.

In various scenarios, EM waves do not approach perpendicularly, but arrive at an angle relative to the MS resonator. Therefore, it is crucial to analyze how different modes of obliquely incident EM waves impact the absorption performance of the MS. The reflection coefficient (Γ) for TE and TM polarization under oblique incidence can be described as follows:

(5) $$\Gamma_{TE}(\omega )=\frac{Z(\omega )\cos\theta_{i}-Z_{o}\cos\theta_{t}}{Z(\omega )\cos\theta_{i}+Z_{o}\cos\theta_{t}}$$

(6) $$\Gamma_{TM}(\omega )=\frac{Z(\omega )\cos\theta_{t}-Z_{o}\cos\theta_{i}}{Z(\omega )\cos\theta_{t}+Z_{o}\cos\theta_{i}}$$

where Z(ω) and $Z_{o}$ are the impedance of the MS resonator and free space, and $\theta_{i}$ and $\theta_{t}$ are the incident and transmission angles, respectively. Based on Equations (5) and (6), the reflection coefficient changes as the incident angle varies. The absorption spectra of the proposed MS harvester were examined by varying the incident angle $\left(\theta_{inc}\right)$ from 0° up to 60° for both TE and TM polarization, as shown in Figure 9. As can be seen in Figure 9a, it is evident that, for TE polarization, the absorption spectrum remained consistent within the range of 0° to 15°, exhibiting an absorption ratio of 98%. When the incident angle increased from 15° to 60°, the absorption peak slightly shifted to a higher frequency. When θ = 30° and θ=45°, the absorption ratio was over 96% at 5.04 GHz and 5.09 GHz, respectively. At θ=60°, an absorption ratio of over 88% was observed at 5.14 GHz. Moreover, when the incident angle changed to θ=75°, the absorption ratio was decreased to 57% at 5.22 GHz. The decrease in absorption can be attributed to the MS absorber’s ability to more easily achieve electrical resonance in response to external EM waves compared to its more challenging magnetic resonance. As the incident angle increased in the TE mode, the magnetic field component along the y-direction gradually decreased, reducing the magnetic flux. This caused a misalignment between the magnetic and electrical resonances, resulting in an impedance mismatch within the MS unit cell and consequently posing challenges in maintaining high absorption levels (See Figure 9a).

For TM polarization, an absorption spectrum over 97% was observed for θ=0° to 30°, as shown in Figure 9b. The absorption peak over 97% and 95% was obtained at 5.1 GHz and 5.2 GHz when θ=45° and 60°, respectively. Furthermore, an absorption peak of 90% was obtained at 5.34 GHz at θ=75°. In the case of the incident TM mode EM wave, it was observed that the magnetic field was consistently aligned with the y-direction. This consistent alignment resulted in a stable response of the MS to the magnetic field, which allowed for strong magnetic resonance at different incident angles, thereby maintaining high levels of absorptance [53].

**Figure 10:**
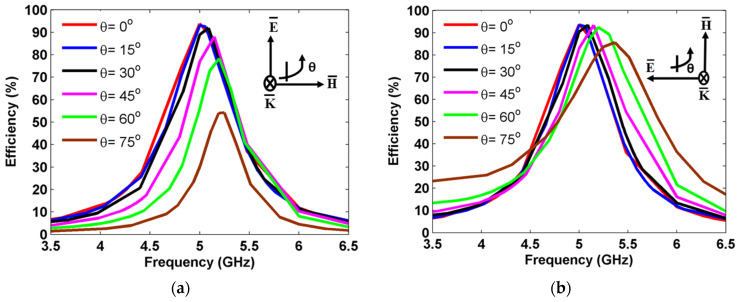
Harvesting efficiency at different incident angles: (**a**) TE polarization and (**b**) TM polarization.

In addition, the harvesting efficiency of the proposed MS unit cell was computed for various incident angles from 0° to 60° for TE and TM polarization, as shown in Figure 10. For TE polarization, a harvesting efficiency peak of about 94% was achieved for the incident angles of 0° to 15°, as shown in Figure 10a. At θ=30°, a harvesting efficiency of over 91% is observed. When the incident angle increased to θ=45° and 60°, the harvesting efficiency decreased to 86% and 74% at 5.15 GHz and 5.2 GHz, respectively. At θ=75°, a harvesting peak of 54% was obtained at 5.19 GHz. In addition, a frequency deviation of 19 MHz was observed as the incident angle increased to 75° for TE polarization. As the incident EM wave’s angle increased in the TE mode, the magnitude of the magnetic field component H in the y-direction decreased progressively. Eventually, sustaining adequate flux to uphold a specific magnetic resonance became challenging. Consequently, the magnetic resonance failed to synchronize with the electrical resonance, resulting in an impedance mismatch within the MS absorber, leading to difficulties in maintaining high efficiency levels (See Figure 10a).

For TM polarization, a 92% harvesting efficiency was obtained at θ=0° as shown in Figure 10b. When the incident angles changed to 15° and 30°, a harvesting efficiency of 93% was observed at 5.08 GHz. A higher harvesting efficiency of over 90% was observed at 5.15 GHz and 5.21 GHz for the incident angles of θ=45° and 60°, respectively. Furthermore, a harvesting efficiency peak of over 85% was achieved at 5.36 GHz when θ=75°. In the case of the TM mode incident EM wave, the magnetic field remained constantly oriented in the y-direction. This alignment ensured a uniform response from the MS to the magnetic field, which allowed for strong magnetic resonance at different angles while maintaining high levels of efficiency.

### 3.2. Experimental Verification

**Figure 11:**
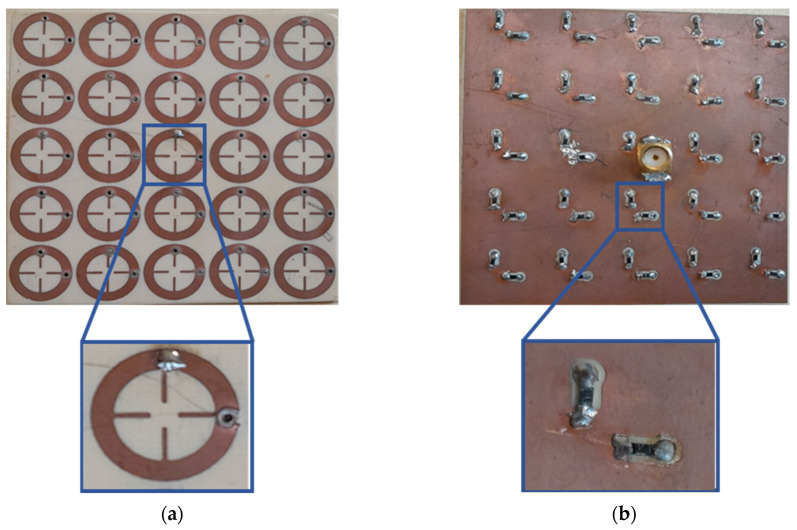
Photo of fabricated MS harvester: (**a**) top view and (**b**) bottom view.

The proposed MS unit cell was initially validated through numerical simulations and tested experimentally by fabricating an MS harvester featuring a 5 × 5 unit cell array. The MS resonator array was printed on a Rogers RO4003C using the printed circuit board (PCB) technique. Each MS resonator was terminated by two ground resistor loads with values of R = 50 Ω, which functioned to mimic the input impedance of a rectifier circuit using two metallic vias with diameters of 1 mm. The central unit cell was connected to one resistor load and VNA connector to measure the conversion efficiency. Figure 11 shows the fabricated MS harvester.

**Figure 12:**
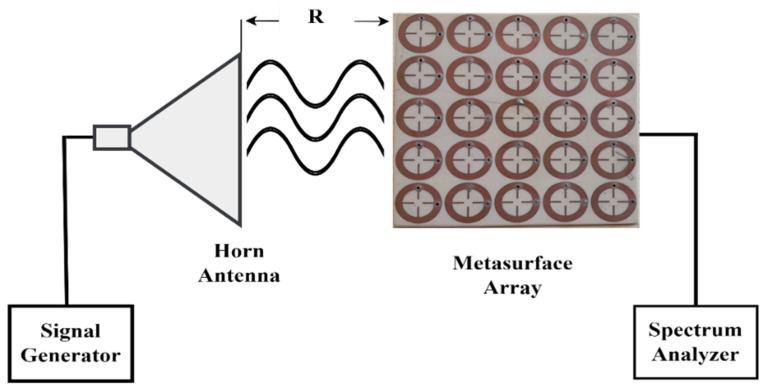
Schematic of the experimental setup.

The experimental measurements were carried out to evaluate the conversion harvesting efficiency $\left(\eta_{RF-AC}\right)$ of the proposed MS harvester, as depicted in Figure 12. The experiment involved using a 5 dBm RF signal generator to transmit signal power to the horn antenna within a specified measurement band. The fabricated MS array was placed in the transmitting horn antenna’s far-field region and connected to a spectrum analyzer to measure the received power. To ensure the optimal excitation of the MS array by the plane wave within the far-field region, it was crucial to meticulously calculate the optimal separation between the transmitting antenna and the MS array. This calculation was achieved using Equation (7), as follows [54]:

(7) $$R>\frac{2D^{2}}{\lambda }$$

where R is the optimal separation between the horn antenna and the MS array, D is the antenna’s aperture, and λ is the wavelength.

The conversion harvesting efficiency was calculated using Equation (4). The power incident $P_{incident}$ into the surface of the MS array was obtained as follows

(8) $$P_{incident}=\frac{G_{t}\cdot P_{t}}{4\pi R^{2}}\times A_{\left(eff,array\right)}$$

where $G_{t}$ is the horn antenna gain; $P_{t}$ is the power excited by the signal generator, which is 5 dBm; and R is the separation distance between the horn antenna and the MS array. In addition, the effective array’s area $A_{\left(eff,array\right)}=25\;A_{eff}$ is equal to the number of the array elements and the effective central unit cell area $\left(A_{eff}\right)$ [47]. The MS structure’s symmetry resulted in the total power received by one of the cells being the sum of the power delivered to both the horizontal and vertical resistors.

**Figure 13:**
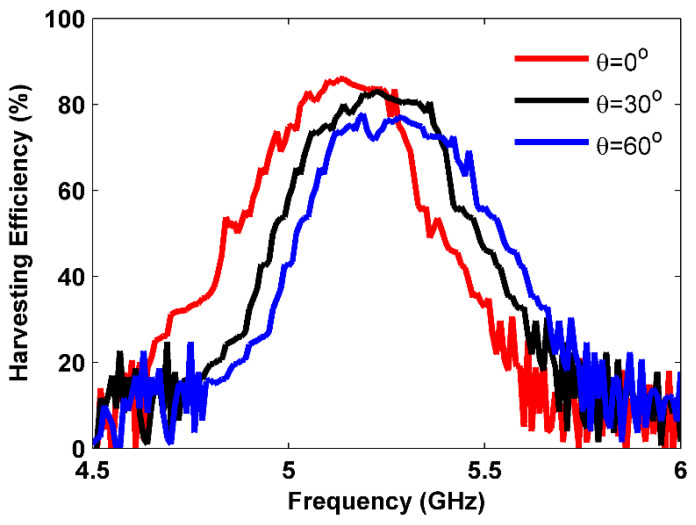
Measured harvesting efficiency at various oblique incidences.

In Figure 13, the measured harvesting efficiency for a 5 × 5 array of the proposed MS harvester is depicted as a function of frequency under various oblique incidence angles. As shown in the figure, higher harvesting efficiencies of over 86% and 83% were observed at 5.13 GHz and 5.22 GHz, respectively, when the incident angles were θ=0° and 30°. At the incident angle of θ=60°, a 77% harvesting efficiency was achieved at 5.28 GHz. Moreover, as the incident angle increased, there was a decrease in the harvesting efficiency and a slight shift in the operating frequency due to the reduction in the magnetic flux. From Figure 13, a slight difference can be observed between the simulation and measurement outcomes in terms of harvesting efficiency. Specifically, the measurement results show a decrease in harvesting efficiency and a slight shift in the operating frequency. This discrepancy occurred because, during the simulation stage, the proposed MS structure was treated as an infinite structure under optimal conditions, whereas, in the measurement stage, the MS unit cell was a finite array. Additionally, fabrication tolerances and the measurement environment further influenced the results.

Table 2 presents a comparison between the proposed MS harvester and similar previous works. The comparison includes the size of the cell, frequency deviation, and harvesting efficiency at the normal and oblique incidence. The harvester proposed in this research features a compact design with higher harvesting frequency over a wide, oblique incident angle with lower frequency deviation than those published earlier. In [25], a compact design was achieved; however, the resistive load was optimized to 3.5 kΩ, which significantly differs from the input impedance of microwave feeding networks, which are crucial components of a rectenna. Consequently, this discrepancy limits the practical application in real-world harvesting systems, making the design of the necessary feeding network using microstrip technology highly challenging and virtually impractical.

## 4. Conclusions

This research presents a polarization-insensitive and wide-angle MS structure that employs a symmetrical wheel resonator for energy harvesting applications. The impedance of the MS cell is adjusted to match that of free space. This innovative MS-based energy harvester can efficiently capture energy from nearby EM waves and convert it into AC power, which can then be transferred to resistive loads through the vias. Simulation results reveal that, when loaded with optimized 50 Ω resistors, the MS harvester absorbs over 98% of normally incident waves, regardless of polarization, and efficiently delivers almost all the absorbed power to the loads. Furthermore, it has been observed that the unit cell is not influenced by polarization when exposed to normal incident plane waves. It achieves a harvesting efficiency of approximately 94% for both TE and TM polarizations under normal incidence, 72% for TE polarization, and 90% for TM polarization at various incident angles up to 60°. A 5 × 5 array of MS cells is fabricated and tested, showing close agreement between measurement and simulation results. It is worth noting that under normal incidence, the measured harvesting efficiency is approximately 86%. Overall, this MS-based energy harvester exhibits promising features, such as high efficiency, polarization insensitivity, and wide-angle coverage, positioning it as a strong contender for energy harvesting applications. Future developments include optimizing the design for multi-band energy harvesting, integrating advanced power management circuits, and exploring flexible materials for wearable and IoT applications.

## Figures and Tables

**Table 1:** Optimized dimensions of the proposed MS unit cell harvester.

Parameters	Value (mm)
Periodicity of the cell (P)	12.7
Outer radius (R1)	6.1
Inner radius R2	3.9
Length of cross-shaped strip (L)	7.73
Width of cross-shaped strip (W)	1
Cross-shaped slot (g)	2.6

**Table 2:** Comparison between the proposed MS structure and similar works.

Ref.	Size	Center Freq. (GHz)	Frequency Deviation	Incident Angle	Efficiency at 0°	Efficiency at 60°
[25]	$0.17\lambda_{o}$	5.8	300 MHz (5.17%)	0°–60°	52%	68%
[43]	$0.29\lambda_{o}$	5.8	48 MHz (0.83%)	0°–30°	91%	72%
[44]	$0.22\lambda_{o}$	7	-	0°–45	95%	N/A
[46]	$0.54\lambda_{o}$	5.4	300 MHz (5.55%)	0°–60°	92%	48%
[47]	$0.65\lambda_{o}$	9.6	-	0°–30°	95%	N/A
[48]	$0.51\lambda_{o}$	5.7	120 MHz (4.44%)	0°–60°	81%	30%
[55]	$0.32\lambda_{o}$	5.8	75 MHz (1.3%)	0°–30°	88%	62%
This work	$0.21\lambda_{o}$	5	19 MHz (0.32%)	0°–60°	94%	76%

## Data Availability

All the data generated during and/or analyzed during the current study are available from the corresponding author upon reasonable request.
